# C16 Phase High Entropy Borides With High Magnetic Anisotropy

**DOI:** 10.1002/adma.202516135

**Published:** 2025-12-23

**Authors:** Willie B. Beeson, Dhritiman Bhattacharya, Dinesh Bista, Bradley J. Fugetta, Gen Yin, Kai Liu

**Affiliations:** ^1^ Physics Department Georgetown University Washington, DC USA

**Keywords:** borides, high entropy materials, high magnetic anisotropy, rare‐earth‐free magnets

## Abstract

High magnetic anisotropy materials have critical applications in numerous technology sectors, largely relying on rare‐earth and precious metals which poses major sustainability challenges. The high entropy composition space offers a vast arena for exploration of high magnetic anisotropy materials based on earth‐abundant elements. However, common high entropy alloys favor disordered cubic crystal structures whereas ordered uniaxial structures are necessary for the desirable strong magnetic anisotropy. Here we report the discovery of novel quinary borides with C16 uniaxial crystal structure and high magnetic anisotropy. Switching the easy‐plane anisotropy of binary C16 borides to easy‐axis can be achieved through a suitable mixing of Fe and Co on the transition metal sublattice. Using a combinatorial sputtering approach, we explore the wider high entropy composition space to further enhance the anisotropy of the C16 phase by incorporation of additional magnetic 3*d* transition metals. Significant coercivity increase, more than two‐fold, has been observed, compared with binary and ternary transition metal borides. Density functional theory calculations support the experimental findings, predicting anisotropy approaching 10^7^ erg/cm^3^, which is understood in terms of the optimized electronic structure of the high entropy borides. These results establish a promising boron‐assisted synthesis strategy to achieve strong magnetic anisotropy using earth‐abundant elements.

## Graphical abstract

Rare‐earth‐free C16‐phase high entropy boride thin films exhibit enhanced magnetic anisotropy with coercivities surpassing their binary and ternary counterparts. Combinatorial synthesis of (Fe‐Co‐Ni‐Mn)_2_B films combined with density functional theory enables mapping of the magnetic properties across the composition space, revealing high entropy compositions with high magnetic anisotropy mediated by an optimized electronic structure.

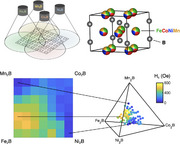

## 1 Introduction

High magnetic anisotropy materials have critical applications in numerous technology sectors, including nanomaterials for magnetic recording media [1, 2, 3, 4, 5, 6, 7, 8, 9] and spintronics [10, 11, 12], as well as high energy density permanent magnets for clean energy technologies [13]. The reliance of these materials on rare‐earth and precious metals is a longstanding concern for sustainability, driving intense effort toward the discovery and fabrication of high anisotropy materials using earth‐abundant elements [14, 15, 16, 17, 18, 19, 20]. However, currently known binary rare‐earth‐free high anisotropy phases, such as MnAl, FeNi, and Fe_16_N_2_, face critical challenges resulting from poor thermodynamic stability and kinetics which have hindered their realization for practical applications [21].

Recently, high entropy alloys (HEAs) have emerged as a platform for materials discovery. Conventional HEAs, defined to contain five or more elements in near‐equiatomic concentrations, exist as stable or metastable single phase solid solutions by virtue of a high configurational entropy [22]. The vast high entropy composition space offers a huge number of unique and unexplored electronic structures, making it an attractive platform for discovery of novel functional materials [23, 24, 25, 26, 27, 28, 29, 30, 31, 32, 33, 34, 35], including high magnetic anisotropy materials [36, 37, 38, 39, 40]. So far, HEA studies have predominantly focused on maximum entropy solid solution phases which generally comprise uniform chemical disorder within cubic crystal structures, while low‐symmetry crystal structure and chemical order play important roles in the magnetocrystalline anisotropy. However, the configurational entropy may be large as long as the number of elements is large relative to the number of unique lattice sites, indicating the possibility to realize stable chemically‐ordered and uniaxial phases which can potentially host high magnetocrystalline anisotropy [36].

Since their inception, HEAs have expanded to include alloys with multiple sublattices, referred to as high entropy intermetallic compounds [36, 41, 42, 43], as well as high entropy oxides, nitrides, and borides which facilitate the formation of sublattices through the combination of metallic and non‐metallic bonding [44]. In particular, the introduction of boron (B) is a promising approach to realize uniaxial and chemically‐ordered HEAs [45, 46]. A potential but previously unexplored high entropy boride phase is the tetragonal C16 phase (space group I4/mcm) which commonly forms in transition metal (TM) borides at 33% B [47, 48]. These compounds are often ferromagnetic with uniaxial magnetic anisotropy due to the tetragonal symmetry of the C16 structure. The ternary C16 (Fe_1−x_Co_x_)_2_B system previously attracted interest for the surprising dependence of its anisotropy on the TM composition [49, 50, 51, 52]. While pure binary Fe_2_B and Co_2_B exhibit low to moderate negative anisotropy constants, undesirable for most high anisotropy applications, the mixing of Fe and Co on the TM sublattice of the C16 structure leads to a switching of the sign of the anisotropy from negative to positive, with a peak value on the order of 10^6^ erg cm^−3^ at the composition (Fe_0.7_Co_0.3_)_2_B [49, 50, 53]. The compositional dependence of the anisotropy primarily arises from the shifting of the Fermi level with respect to coupled spin states [50]. Considering the sensitive dependence of the anisotropy on the parameters of individual TMs, this system is a compelling platform for extension to the high entropy parameter space in search of further enhanced anisotropy. In this study, we introduce additional 3*d* TMs to realize C16 phase high entropy borides and explore their properties across the composition space using a combinatorial co‐sputtering method, leading to the discovery of novel quinary boride compositions with high magnetic anisotropy.

## 2 Results and Discussion

Prior to fabrication of high entropy films, Fe_2_B and (Fe_1−_ *_x_* Co*_x_*)_2_B films were grown on Si/SiO_2_ substrates by sputtering and rapid thermal annealing (RTA) as discussed in Methods to confirm the formation of the C16 structure and magnetic properties. Figure S2 shows the X‐ray diffraction (XRD) scans of 20 nm Fe_2_B and (Fe_0.7_Co_0.3_)_2_B films as‐grown and after RTA treatment for 120 s at 600°C. The as‐grown films were found to be amorphous, while the RTA‐treated films exhibit characteristic C16 diffraction peaks with comparable lattice parameters to bulk [52, 54]. A series of 50 nm (Fe_1−_ *_x_* Co*_x_*)_2_B films with varying Co concentration were fabricated by co‐sputtering and treated with RTA at 600°C for 240 s. The coercivity *H_c_* was measured as a function of Co concentration at 300 K and the trend was found to correspond with that of *K_u_* of bulk C16 (Fe_1−x_Co_x_)_2_B at 300 K [53], as shown in Figure S3. Specifically, the *H_c_* exhibits local minima near the expected spin reorientation transitions, and a local maximum near the expected maximum in *K_u_*. Thus, we show that the trends in *H_c_* with respect to the transition metal composition of the boride films largely reflect the variation in magnetocrystalline anisotropy of the C16 phase.

**FIGURE 1:**
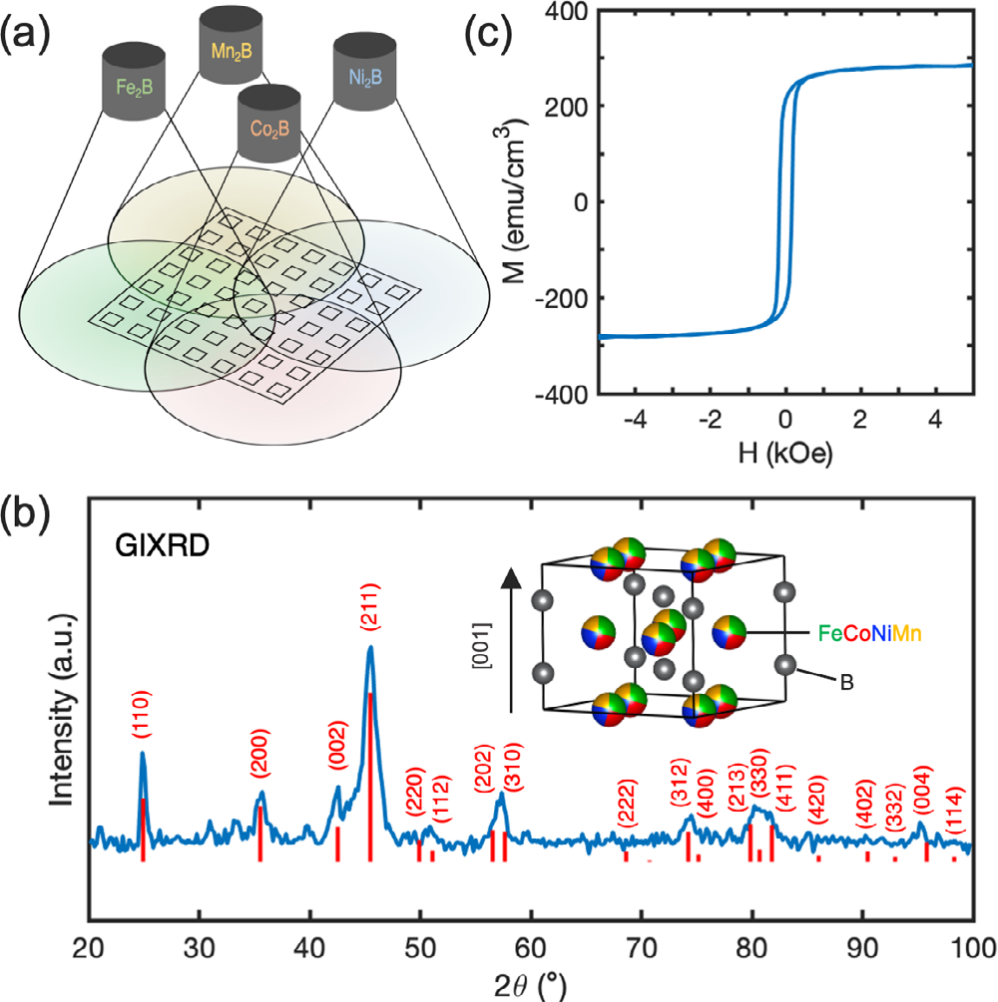
(a) Schematic of combinatorial sputtering, showing 4 sputtering targets positioned in a square layout and centered over the substrate. The substrate is masked into a 7 × 7 square grid. (b) GIXRD (ω = 1°) of a 50 nm (FeCoNiMn)_2_B film treated with RTA for 120 s. The red lines are the calculated powder diffraction peak for a C16 structure with lattice parameters of *a* = 5.05 Å and *c* = 4.25 Å. The inset shows a schematic illustration of the C16 crystal structure. (c) In‐plane hysteresis loop of the equimolar (FeCoNiMn)_2_B film at 300 K.

Extending to the high entropy system, we introduced Ni and Mn to form a quinary (Fe─Co─Ni─Mn)_2_B film by co‐sputtering of Fe_2_B, Co_2_B, Ni_2_B, and Mn_2_B targets, as discussed in Methods and schematically shown in Figure 1a. We first targeted the equimolar composition (FeCoNiMn)_2_B by co‐sputtering with substrate rotation on. A composition of (Fe_0.30_Co_0.23_Ni_0.26_Mn_0.21_)_2_B with near‐equiatomic ratio of TMs was determined by energy dispersive X‐ray microanalysis (EDX), which satisfies the high entropy intermetallic phase formation rules with atomic size difference δ*r* = 19% and overall electronegativity difference *η* = 0.25 [42], assuming a nominal 1:2 ratio of B to TMs. Figure 1b shows the grazing incidence X‐ray diffraction (GIXRD) scans (ω = 1°) of 50 nm (FeCoNiMn)_2_B films treated with RTA at 600°C for 120 s. The films were found to crystallize into a C16 phase with lattice parameters of *a* = 5.05 Å and *c* = 4.25 Å. The calculated powder diffraction pattern for this structure is displayed in Figure 1b and can be seen to well‐match the measured diffraction profile, confirming C16 phase formation in the high entropy film. The agreement in relative intensities of the GIXRD peaks with the simulated diffraction pattern indicates a polycrystalline film with random grain orientation. Additional unindexed peaks can be seen in the range of 2θ < 40°, which likely originate from a secondary phase. The peaks at 21°, 31°, and 33° are consistent with the major peaks of borate phases Mn_2_BO_4_ and Fe_2_BO_4_ [55, 56]. Given that these phase have the same TM:B ratio as the film, they are considered as possible secondary phases, which would indicate some oxidation of the film during RTA. Figure 1c shows the in‐plane hysteresis loop of the (FeCoNiMn)_2_B film, which exhibits a low *M_s_* = 284 emu cm^−3^ and a modest *H_c_* = 166 Oe. The low *H_c_* compared with that of the 50 nm (Fe_0.7_Co_0.3_)_2_B film (*H_c_* = 410 Oe) suggests a relatively low anisotropy in the equimolar C16 high entropy boride.

To explore the magnetic properties of the (Fe─Co─Ni─Mn)_2_B system as a function of TM composition, two series of samples (labeled Series 1 and Series 2) were deposited by combinatorial sputtering as described in Methods and treated with RTA at 600°C for 60 s. Samples are labeled by their series and position in the grid (rows A‐G and columns 1–7). The EDX‐determined compositions for all samples are listed in Table S1, which assume a nominal 1:2 B:TM ratio equal to the stoichiometric X_2_B targets used for deposition. The atomic ratios of each TM are similarly distributed throughout the range of ∼10% to ∼50% of the total TM concentration, as illustrated in Figure S1. The mean thickness of each series was 22 nm as determined by X‐ray reflectivity (XRR).

**FIGURE 2:**
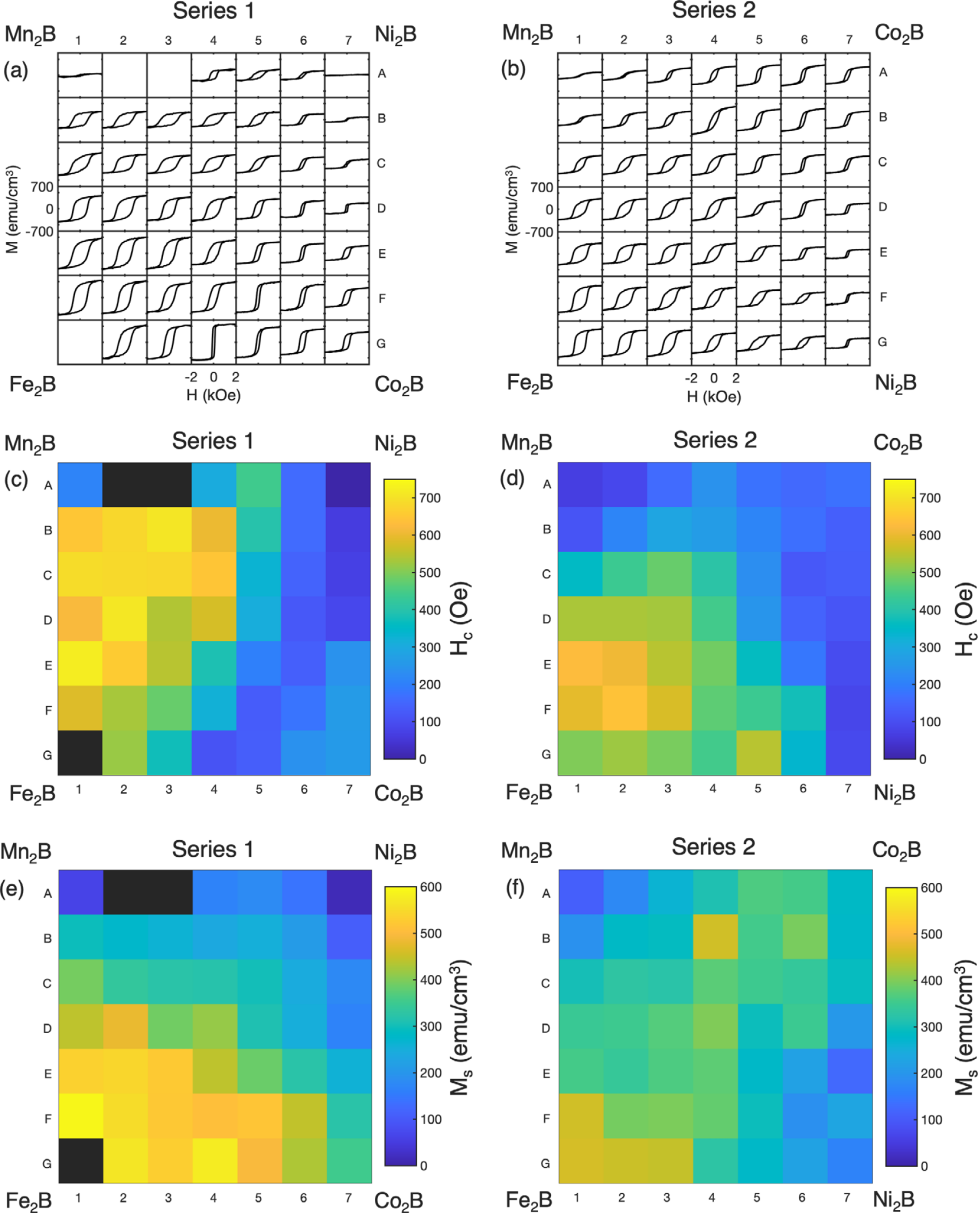
(a,b) Room temperature hysteresis loops and corresponding heat maps of (c,d) *H_c_* and (e,f) *M_s_* for two combinatorial (Fe_x_Co_y_Ni_z_Mn_1−x−y−z_)_2_B series 1 and 2 after RTA at 600°C for 60 s.

**FIGURE 3:**
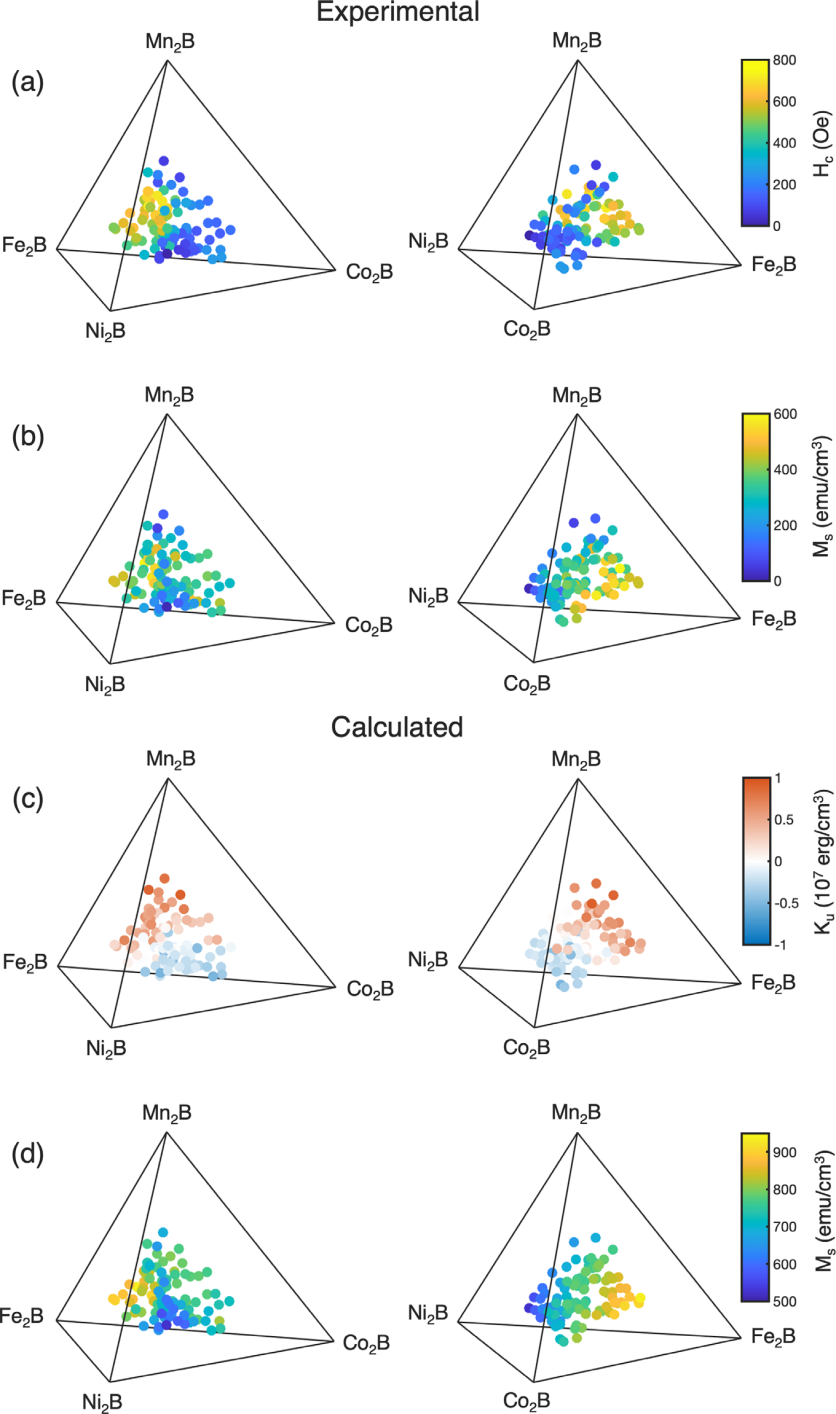
Quaternary plots of experimentally measured (a) *H_c_* and (b) *M_s_* of combinatorial (Fe_x_Co_y_Ni_z_Mn_1−x−y−z_)_2_B films and DFT calculated (c) *K_u_* and (d) *M_s_* for a C16 phase with the compositions listed in Table S1. The left and right column show two different views of the quaternary plot.

In‐plane hysteresis loops for the combinatorial samples were collected at room‐temperature using vibrating sample magnetometry (VSM), as shown in Figure 2a,b. Heat maps of the coercivity *H_c_* and saturation magnetization *M_s_* for both series are shown in Figure 2c–f. Samples 1‐A2, 1‐A3, and 1‐G1 are omitted due to oxidation of the sample during annealing. In both series, the samples with the highest *H_c_* are located at off‐corner cells of the grid. Specifically, regions of high *H_c_* > 600 Oe are found on the Fe─Mn‐rich side of both series. The sample with the highest *H_c_* overall was sample 1‐E1 with composition (Fe_0.35_Co_0.17_Ni_0.17_Mn_0.31_)_2_B, exhibiting *H_c_* = 720 Oe and *M_s_* = 536 emu cm^−3^. The sample with highest *H_c_* in Series 2 was sample 2‐F2 with composition (Fe_0.46_Co_0.16_Ni_0.19_Mn_0.19_)_2_B, *H_c_* = 640 Oe and *M_s_* = 393 emu cm^−3^. Meanwhile, the highest *M_s_* was found for sample 1‐F1 with composition (Fe_0.41_Co_0.18_Ni_0.14_Mn_0.27_)_2_B, exhibiting *M_s_* = 591 emu cm^−3^ and *H_c_* = 580 Oe. Both Co‐rich and especially Fe‐rich samples generally exhibit higher magnetizations, consistent with the relatively high moments of 1.89 μ_*B*_ and 0.80 μ_*B*_ for Fe and Co in Fe_2_B and Co_2_B, respectively [57]. Meanwhile, a discernible trend is the reduction in magnetization at higher concentrations of either Ni or Mn. This is consistent with the reported paramagnetic and antiferromagnetic ordering of Ni_2_B and Mn_2_B, respectively [58]. The sample with the lowest *M_s_* and *H_c_* was sample 1‐A7 with Ni‐rich composition (Fe_0.12_Co_0.24_Ni_0.48_Mn_0.16_)_2_B, and was the only sample with no ferromagnetic signal at room‐temperature. Notably, this composition has the highest Ni:Fe ratio of all samples. The sample with the second lowest *M_s_* was sample 1‐A1 with Mn‐rich composition of (Fe_0.17_Co_0.13_Ni_0.22_Mn_0.48_)_2_B, *H_c_* = 210 Oe and *M_s_* = 62 emu cm^−3^. While both Ni and Mn substantially reduce *M_s_* at high concentrations, it appears that a moderate concentration of Mn is effective in enhancing *H_c_*, being the metal with second highest concentration in the sample with highest measured *H_c_* (1‐E1). We note that the coercivity values observed here significantly exceed those previously reported for ternary (Fe_0.7_Co_0.3_)_2_B (380 Oe [59], 350 Oe [60]) as well as that found for (Fe_0.7_Co_0.3_)_2_B in the present study (410 Oe, Figure S3). For better visualization of the trends in magnetic properties throughout the composition space, the *H_c_* and *M_s_* values for all samples are plotted in quaternary diagrams (Figure 3) based on the EDX‐determined TM concentrations listed in Table S1.

XRD was carried out on selected samples for phase analysis (Figure S4). All measured samples exhibit characteristic C16 diffraction peaks, confirming the formation of C16 phase across the combinatorial film. Alongside the C16 phase, Ni‐rich and Co‐rich samples exhibit diffraction peaks consistent with a D8_4_ phase. Such boride phases (e.g., Fe_23_B_6_, Co_23_B_6_, Ni_23_B_6_) are metastable and typically observed as secondary crystallization products during rapid solidification [61, 62]. We note that the presence of D8_4_ phases, as well as any residual amorphous phase, may lead to deviations in the measured magnetic properties from that of the pure C16 phase. For example, D8_4_ Fe_23_B_6_ has been reported to exhibit *M_s_* between 153 emu/g and 196 emu/g depending on stoichiometry, compared to 157 emu/g reported for C16 Fe2B [63, 64]. Additionally, the D8_4_ phases are cubic, thus are expected to reduce the total anisotropy, consistent with the low *H_c_* of samples 1‐G7, 2‐A7, and 2‐G7 seen in Figure 2d. Certain samples (e.g., 2‐A1) also exhibit low *H_c_* despite the dominant C16 phase, thus the D8_4_ and C16 phase fractions cannot account for the variation in magnetic properties alone. Despite the presence of soft cubic and/or amorphous parent phases, we expect the C16 phase seen in XRD to predominantly contribute to the *H_c_* by serving as magnetically hard domain wall pinning sites. The degree to which these enhance the coercivity depends on the specific microstructure as well as the compositional dependence of the C16 phase magnetocrystalline anisotropy. We also note the presence of film voids and discontinuities (Figure S5) which may further contribute to the observed coercivity, as discussed in Supporting Information.

To investigate the composition‐dependent variations in magnetic properties within the C16 phase, excluding microstructural factors, density functional theory (DFT) calculations using coherent potential approximation (CPA) were carried out for the 98 sample compositions determined via EDX, assuming a C16 structure with the lattice parameters of the equimolar (FeCoNiMn)_2_B film (Figure 1). Figure 3c,d show quaternary plots of the DFT‐calculated *K_u_* and *M_s_*, respectively. Despite variations in film microstructure, the trends in *K_u_* and *M_s_* match well with those in the experimentally measured *H_c_* and *M_s_* (Figure 3a,b, respectively). DFT predicts high positive *K_u_* approaching 1 × 10^7^ erg cm^−3^ on the (Fe,Mn)‐rich side of the composition space, with a spin reorientation transition to negative *K_u_* on the (Co,Ni)‐rich side. The region of high experimental *H_c_* (> 400 Oe) is contained within the region of positive *K_u_* predicted by DFT. Thus, the high *H_c_* of these samples can be attributed in part to the formation of C16 phase with high positive *K_u_*. Meanwhile, the majority of low *H_c_* samples (*H_c_* < 300 Oe) are found in the region of low negative *K_u_* predicted by DFT, which does not fall below −5 × 10^6^ erg cm^−3^. We note that the presence of the soft D8_4_ phase in Co‐rich and Ni‐rich samples will further reduce the anisotropy. The crossing of *K_u_* through 0 near the center of the composition space may explain the low *H_c_* of equimolar (FeCoNiMn)_2_B films shown in Figure 1c.

**FIGURE 4:**
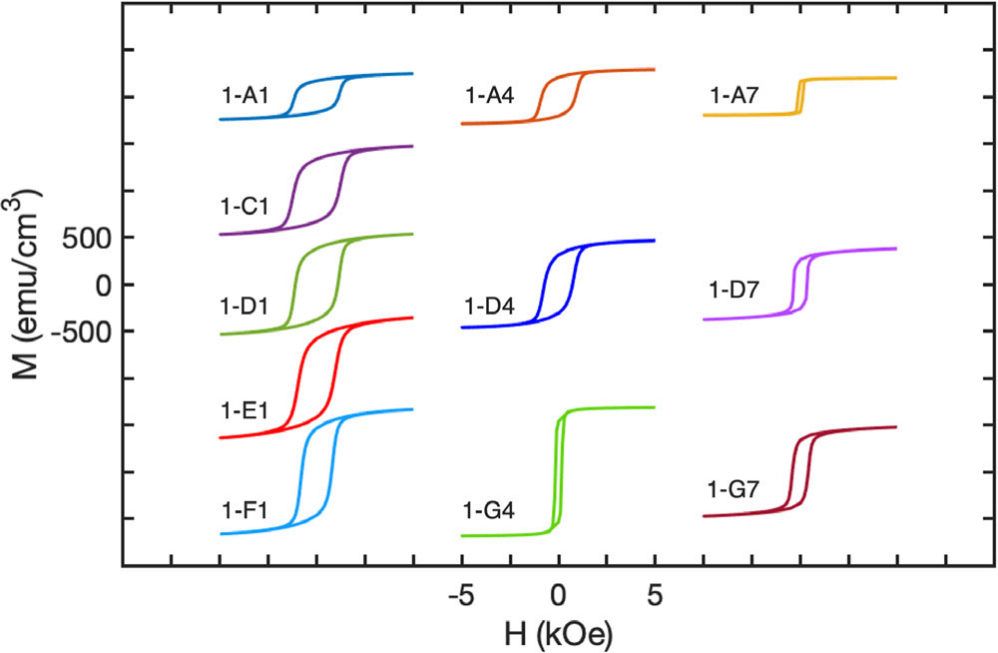
Hysteresis loops of combinatorial (Fe_x_Co_y_Ni_z_Mn_1−x−y−z_)_2_B samples measured at 5 K for the in‐plane geometry.

Several of the samples with Mn‐rich compositions exhibit small coercivity experimentally, despite a high positive anisotropy predicted by DFT at 0 K. For example, sample 1‐A1 with composition (Fe_0.17_Co_0.13_Ni_0.22_Mn_0.48_)_2_B is predicted to have a sizeable *K_u_* of 6.2 × 10^6^ erg cm^−3^, while exhibiting a relatively low *H_c_* of 210 Oe, despite the dominant C16 phase seen in XRD. To investigate the role of temperature in this discrepancy, we carried out hysteresis loop measurements on select samples at 5 K via superconducting quantum interference device (SQUID) magnetometry, as shown in Figure 4, as well as temperature‐dependent magnetization measurements (Figure S8). Sample 1‐A1 exhibits a dramatic increase in *H_c_* from 210 Oe at 300 K to 1130 Oe at 5 K, along with an increase in *M_s_* from 62 to 323 emu cm^−3^. This suggests that a low Curie temperature in this sample results in low *K_u_* at room‐temperature and correspondingly a low *H_c_*. Meanwhile, the large *H_c_* at 5 K is consistent with the high *K_u_* predicted at 0 K by DFT. Samples 1‐A7 and 1‐D7 with compositions (Fe_0.12_Co_0.24_Ni_0.48_Mn_0.16_)_2_B and (Fe_0.14_Co_0.32_Ni_0.36_Mn_0.18_)_2_B also exhibit low Curie temperatures; however, in contrast to 1‐A1, their *H_c_* remains relatively low at 5 K (120 Oe and 350 Oe, respectively), consistent with their moderate negative *K_u_* values predicted by DFT. Thus, the discrepancy between measured *H_c_* and calculated *K_u_* in the Mn‐rich region can be attributed primarily to the ambient temperature of the experimental measurements. Besides 1‐A1, 1‐A7, and 1‐D7, all other samples showed FM behavior throughout the measurement range of 5 to 375 K. Therefore, we find that the thermal stability of the FM phase is highly sensitive to the TM composition, with high Fe concentrations enhancing the Curie temperature and high Ni or Mn concentrations reducing it substantially. Notably, the sample with the largest *H_c_* at 5 K was sample 1‐C1 with composition (Fe_0.29_Co_0.14_Ni_0.19_Mn_0.38_)_2_B, exhibiting *H_c_* = 1160 Oe and *M_s_* = 577 emu cm^−3^, with *K_u_* of 5.6 × 10^6^ erg cm^−3^ predicted by DFT.

Despite the temperature effects discussed above, as well as the presence of additional phases, there is good qualitative agreement between the trends in *M_s_* obtained in experiment (Figure 3b) and theory (Figure 3d). Consistent with experiment, DFT calculations show especially high *M_s_* in Fe‐rich compositions and low *M_s_* in Ni‐rich compositions. As shown in Figure S6, the calculated and experimental *M_s_* are linearly correlated with a slope of ∼1 and offset of 536 emu cm^−3^. The offset may be attributed in part to the temperature difference as well as inherent overestimation by the CPA method.

**FIGURE 5:**
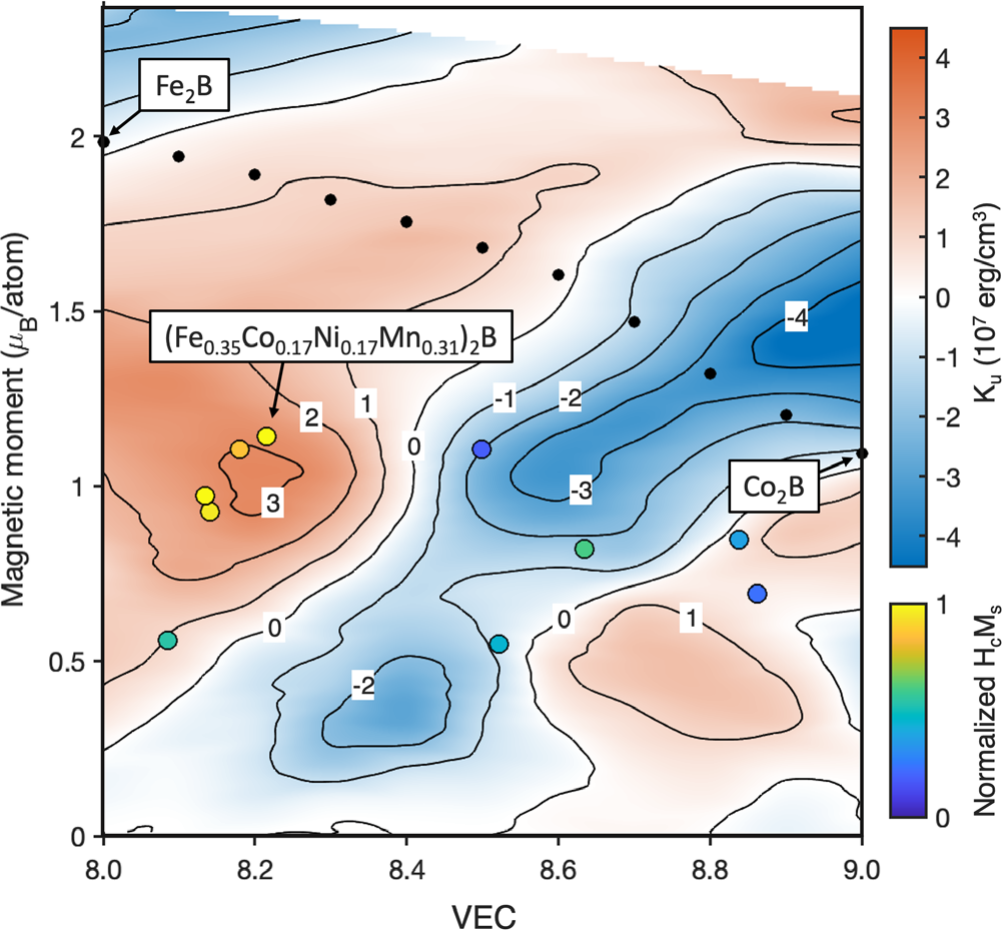
Map of the calculated magnetic anisotropy *K_u_* in (Fe_1−x_Co_x_)_2_B as a function of the VEC and average moment of TM species, replotted from Ref. [52]. The black circles are the values calculated for (Fe_1−x_Co_x_)_2_B [52]. The colored circles represent selected (Fe_x_Co_y_Ni_z_Mn_1−x−y−z_)_2_B samples measured at 5 K in the present work. The average TM moment is estimated from the *M_s_* extracted via SQUID and VEC from the TM composition extracted via EDX. The circles are colored according to the product of *H_c_* and *M_s_* measured at 5 K.

The high anisotropy of the high entropy C16 borides can be understood in terms of the optimization of the electronic structure. The compositional dependence of the anisotropy in (Fe_1−x_Co_x_)_2_B primarily arises from the shift in Fermi level with respect to coupled minority spin states [50]. This is determined both by the exchange splitting, related to the magnetic moment, and the effective number of valence electrons which changes with Co concentration. Figure 5 shows a map of the theoretical *K_u_* for (Fe_1−x_Co_x_)_2_B as a function of the valence electron concentration (VEC) and average moment on the TM site, replotted from Edström et al. [52]. The black circles represent the calculated values for (Fe_1−x_Co_x_)_2_B from Ref. [52]. In (Fe_1−x_Co_x_)_2_B, the optimal band‐filling for high positive *K_u_* is achieved at a TM VEC between 8.3 and 8.4, corresponding to a Co concentration of *x* = 0.3 [49, 50, 51, 52]. The map of Figure 5 reveals the wider energy landscape on which the ternary (Fe_1−x_Co_x_)_2_B exists. Interestingly, a peak in *K_u_* of over 3 × 10^7^ erg cm^−3^ is predicted at VEC ≈ 8.2 for a moment of ∼1 μ_*B*_ [52]. While Ref. [52] notes that the treatment of compositional disorder by virtual crystal approximation tends to overestimate the anisotropy overall, the qualitative trend along the (Fe_1−x_Co_x_)_2_B path matches well with that of experiment [53], supporting the potential for further enhanced anisotropy by tuning of the VEC and average moment. As pointed out by Ref. [52], satisfying both of the conditions with a single dopant starting from (Fe_0.6_Co_0.4_)_2_B would require the introduction of expensive 4*d* or 5*d* metals.

To compare the (Fe_x_Co_y_Ni_z_Mn_1−x−y−z_)_2_B samples in the present work with the calculations of Ref. [52], the effective TM moment and VEC were estimated for the samples measured at 5 K (Figure 4). The effective TM moment is estimated from *M_s_* measured in SQUID at 5 K using the C16 lattice parameters of the equimolar sample (Figure 1b). A small negative boron contribution was subtracted from the total moment per unit cell, estimated as the composition‐weighted average of reported boron moments in Fe_2_B, Co_2_B, Ni_2_B and Mn_2_B [58]. The VEC was calculated based on the TM compositions listed in Table S1, as described in Methods. The colored circles in Figure 5 denote the samples’ positions in the map. For illustration, the circles are colored according to the product of *H_c_* and *M_s_* measured at 5 K, normalized to the maximum value. We use this product as a qualitative proxy of the magnetic anisotropy since *H_c_* ∝*K_u_*/*M_s_*, assuming no effective demagnetization factor in the plane of the film.

The samples with the highest *H_c_M_s_* are samples 1‐C1, 1‐D1, 1‐E1 and 1‐F1 with compositions of (Fe_0.29_Co_0.14_Ni_0.19_Mn_0.38_)_2_B, (Fe_0.36_Co_0.18_Ni_0.14_Mn_0.32_)_2_B, (Fe_0.35_Co_0.17_Ni_0.17_Mn_0.31_)_2_B, and (Fe_0.41_Co_0.18_Ni_0.14_Mn_0.27_)_2_B, respectively. Notably, these samples are all located in the region of the map where *K_u_* > 2 × 10^7^ erg cm^−3^. The sample composition (Fe_0.35_Co_0.17_Ni_0.17_Mn_0.31_)_2_B with the highest room‐temperature coercivity exhibits a VEC of 8.22 and effective moment of 1.1 μ_*B*_, close to the optimal values predicted by [52]. The mixing of Ni and Mn onto the TM sublattice with Fe and Co allows a reduction in the effective moment due to the reduced Ni and Mn moments, meanwhile the VEC can be maintained in the optimal region by the Ni:Mn ratio due to the valence electron numbers of 10 for Ni and 7 for Mn. While low‐temperature measurements were not conducted on all samples for comparison with the map of Figure 5, it is illustrative to examine the distribution of VEC for all combinatorial samples, which is found to be in the range of 7.8 to 9.1 as shown in Figure S7. The bins are colored according to the samples’ normalized *H_c_M_s_* measured at 300 K. The samples with high *H_c_M_s_* are concentrated in the range of 8.1< VEC < 8.4, which aligns with the region of highest positive *K_u_* in Figure 5. Thus, we suggest that the high anisotropy in C16 high entropy borides is due to an optimized electronic structure achieved through simultaneous tuning of the 3*d* VEC and effective moment. Although the random polycrystalline microstructure limits accurate determination of the anisotropy constants, effective anisotropy constants *K*_eff_ at 5 K were estimated for samples 1‐C1, 1‐D1, 1‐E1 and 1‐F1 by fitting their high‐field magnetization curves to the law of approach to saturation $M=M_{s}\;\;-\frac{A}{H^{2}}$ where $A=\frac{4}{15}\;\frac{{K_{u}}^{2}}{M_{s}}$ for randomly oriented uniaxial crystallites [65]. A linear fitting of *M* vs. 1/*H* ^2^ from 4 to 7 T yields *K*_eff_ values of approximately 8 × 10^6^ erg/cm^3^ for 1‐C1, 6 × 10^6^ erg/cm^3^ for 1‐D1, 7 × 10^6^ erg/cm^3^ for 1‐E1, and 6 × 10^6^ erg/cm^3^ for 1‐F1.

## 3 Conclusion

In summary, we have demonstrated high magnetic anisotropy in novel rare‐earth‐free and precious‐metal‐free C16‐ordered high entropy boride films. Equimolar (FeCoNiMn)_2_B films exhibit clear C16 chemical ordering after RTA as shown by XRD. While the equimolar films exhibit ferromagnetic properties, their anisotropy is modest. To optimize the anisotropy in the wider composition space, we employed a combinatorial fabrication process and mapped the magnetic properties as a function of the transition metal composition. This led to the discovery of novel boride compositions with high magnetic anisotropy and coercivity up to 720 Oe at room‐temperature and 1.2 kOe at 5 K, which was supported by DFT calculations based on CPA predicting anisotropy up to 1 × 10^7^ erg cm^−3^. Although measurements of the anisotropy constants in single‐crystal high entropy borides are needed for more accurate comparison, the values estimated from polycrystalline samples as well as those predicted by DFT both exceed the maximum anisotropy of 5 × 10^6^ erg cm^−3^ reported for ternary (Fe_1−x_Co_x_)_2_B [53], indicating the potential for significantly enhanced anisotropy in the high entropy system. These results are consistent with previous theoretical investigations predicting increased anisotropy for specific values of the effective moment and valence electron concentration [52]. While prior experiments have demonstrated anisotropy enhancement via introduction of rare 5*d* metals [60, 66], our work further demonstrates the parameter optimization with comparable property enhancement using only abundant 3*d* metals by leveraging the unique tunability of the high entropy system. Such high entropy borides could be well‐suited for applications which require more moderate or low magnetization in combination with high anisotropy, such as heat‐assisted magnetic recording media or magnetic tunnel junctions, while also opening up a new avenue for sustainable permanent magnet explorations. More broadly, these studies highlight the rich energy landscape of an ordered high entropy material system, motivating future exploration of other phases for exceptional magnetic properties.

## 4 Experimental Section

All films were deposited by DC magnetron sputtering in an ultrahigh vacuum system with a base pressure of ∼1 × 10^−8^ Torr. Multicomponent boride films were deposited by co‐sputtering of stoichiometric boride targets (Fe_2_B, Co_2_B, Ni_2_B, Mn_2_B) onto thermally oxidized Si(100) substrates with a 200 nm thick amorphous SiO_2_ layer. All depositions were performed at room‐temperature with an Ar working pressure of 5 mTorr. Films were capped with a 2.5 nm thick layer of Ta to protect from oxidation. After deposition, films were treated with rapid thermal annealing (RTA) at 600°C in a vacuum chamber with base pressure ∼1 × 10^−8^ Torr for 60–120 s.

For combinatorial deposition, a 4″ diameter Si/SiO_2_ wafer was masked into a 7 × 7 grid of 5 × 5 mm^2^ cells. The Fe_2_B, Co_2_B, Ni_2_B and Mn_2_B targets were positioned in the chamber in a square layout centered over the substrate, as depicted in Figure 1a. The substrate was fixed (non‐rotating) during deposition to allow the formation of a composition gradient across the film according to the cells’ distance from each target. The relative positions of the sputtering targets were permutated to produce two series of combinatorial films with distinct composition profiles. For Series one, the clockwise sequence of targets was Fe_2_B, Mn_2_B, Ni_2_B, Co_2_B. For Series 2, the sequence was Fe_2_B, Mn_2_B, Co_2_B, Ni_2_B. For films sputtered with a rotating substrate, the transition metal compositions were controlled by adjusting the target sputtering powers and/or gun tilt angles.

X‐ray diffraction with Cu K_α_ radiation was employed for structural characterization using a Panalytical X'Pert^3^ Materials Research Diffractometer. Diffraction intensities are calculated using the absorption factor for grazing‐incidence geometry and powder Lorentz factor, in addition to the structure factors, multiplicity, Debye‐Waller factor, and polarization factor. Surface topography was imaged using a Zeiss SUPRA 55‐VP scanning electron microscope. An Oxford Instruments Energy‐Dispersive X‐ray Microanalysis system was used to analyze chemical composition of the films. The TM concentrations are expected to be accurate to within ± 3 at.%. The boron concentration was not quantified by EDX due to the inaccuracy of light element quantification. We state a nominal 1:2 B:TM ratio corresponding to that of the stoichiometric X_2_B sputtering targets. Film thicknesses were determined by X‐ray reflectivity (XRR). The mean thicknesses of both combinatorial films were 22 nm with <10% variation across the grid.

Room‐temperature magnetic measurements were performed via vibrating sample magnetometry (VSM) using a Princeton Measurements Corporation MicroMag. Low temperature measurements were performed via superconducting quantum interference device (SQUID) magnetometry in a Quantum Design Magnetic Property Measurements System (MPMS3) system.

First‐principles calculations were conducted within the DFT framework, utilizing the Green's function approach alongside the linear muffin‐tin orbitals (LMTO) method under the atomic sphere approximation (ASA). To model compositional disorder in HEAs, spin‐polarized self‐consistent calculations employed the coherent potential approximation (CPA) [67] as implemented in the ‘lmgf’ package within the Questaal suite [68].

The valence electron concentration (VEC) refers to the effective number of electrons, including *d* electrons, accommodated in the valence band. This was calculated for according to VEC = $\sum_{i}^{N}x_{i}\left(\mathrm{VE}C_{i}\right)$ where VEC_*i*_ is the integer number of valence electrons of the *i* ^th^ element and *x_i_* is the atomic concentration of the *i* ^th^ element determined via EDX (Table S1) [69].

## Conflicts of Interest

W.B. and K.L. are co‐inventors on a pending patent application on Boron‐based and high entropy magnetic materials filed by Georgetown University.

## Supporting information




**Supporting file**: adma71813‐sup‐0001‐SuppMat.docx.

## Data Availability

The data that support the findings of this study are available in the supplementary material of this article.
