# Supplementary material for: C16 Phase High Entropy Borides With High Magnetic Anisotropy
Source: Adv Mater. 2025 Dec 23;38(22):e16135. doi: 10.1002/adma.202516135 (PMC13088217; doi:10.1002/adma.202516135)
Supplement: Supplementary file 1 — Supporting file: adma71813‐sup‐0001‐SuppMat.docx. [file ADMA-38-e16135-s001.pdf]

Supporting Information

C16 Phase High Entropy Borides with High Magnetic Anisotropy

Willie B. Beeson, Dhritiman Bhattacharya, Dinesh Bista, Bradley J. Fugetta Gen Yin, Kai Liu**

Physics Department, Georgetown University, Washington, DC 200057, USA

Table S1. Compositions of combinatorial $(Fe_xCo_yNi_zMn_{1-x-y-z})_2B$ samples estimated by EDX. The atomic ratio of B is nominal and was not analyzed by EDX.

Series 1

	1	2	3	4	5	6	7
A	$(Fe_{0.17}Co_{0.13}Ni_{0.22}Mn_{0.48})_2B$	$(Fe_{0.22}Co_{0.13}Ni_{0.26}Mn_{0.39})_2B$	$(Fe_{0.18}Co_{0.18}Ni_{0.27}Mn_{0.37})_2B$	$(Fe_{0.14}Co_{0.19}Ni_{0.34}Mn_{0.33})_2B$	$(Fe_{0.13}Co_{0.22}Ni_{0.39}Mn_{0.26})_2B$	$(Fe_{0.13}Co_{0.21}Ni_{0.45}Mn_{0.21})_2B$	$(Fe_{0.12}Co_{0.24}Ni_{0.48}Mn_{0.16})_2B$
B	$(Fe_{0.26}Co_{0.13}Ni_{0.22}Mn_{0.39})_2B$	$(Fe_{0.27}Co_{0.14}Ni_{0.23}Mn_{0.36})_2B$	$(Fe_{0.26}Co_{0.18}Ni_{0.26}Mn_{0.30})_2B$	$(Fe_{0.19}Co_{0.19}Ni_{0.33}Mn_{0.33})_2B$	$(Fe_{0.17}Co_{0.26}Ni_{0.40}Mn_{0.17})_2B$	$(Fe_{0.13}Co_{0.26}Ni_{0.39}Mn_{0.22})_2B$	$(Fe_{0.12}Co_{0.29}Ni_{0.41}Mn_{0.17})_2B$
C	$(Fe_{0.29}Co_{0.14}Ni_{0.19}Mn_{0.38})_2B$	$(Fe_{0.26}Co_{0.22}Ni_{0.22}Mn_{0.30})_2B$	$(Fe_{0.23}Co_{0.23}Ni_{0.23}Mn_{0.31})_2B$	$(Fe_{0.21}Co_{0.21}Ni_{0.32}Mn_{0.29})_2B$	$(Fe_{0.14}Co_{0.24}Ni_{0.38}Mn_{0.24})_2B$	$(Fe_{0.18}Co_{0.27}Ni_{0.37}Mn_{0.18})_2B$	$(Fe_{0.14}Co_{0.34}Ni_{0.38}Mn_{0.14})_2B$
D	$(Fe_{0.36}Co_{0.18}Ni_{0.14}Mn_{0.32})_2B$	$(Fe_{0.27}Co_{0.18}Ni_{0.18}Mn_{0.37})_2B$	$(Fe_{0.24}Co_{0.24}Ni_{0.24}Mn_{0.28})_2B$	$(Fe_{0.23}Co_{0.23}Ni_{0.31}Mn_{0.23})_2B$	$(Fe_{0.18}Co_{0.27}Ni_{0.32}Mn_{0.23})_2B$	$(Fe_{0.17}Co_{0.31}Ni_{0.30}Mn_{0.22})_2B$	$(Fe_{0.14}Co_{0.32}Ni_{0.36}Mn_{0.18})_2B$
E	$(Fe_{0.35}Co_{0.17}Ni_{0.17}Mn_{0.31})_2B$	$(Fe_{0.35}Co_{0.22}Ni_{0.17}Mn_{0.26})_2B$	$(Fe_{0.38}Co_{0.24}Ni_{0.14}Mn_{0.24})_2B$	$(Fe_{0.25}Co_{0.29}Ni_{0.25}Mn_{0.20})_2B$	$(Fe_{0.25}Co_{0.35}Ni_{0.20}Mn_{0.20})_2B$	$(Fe_{0.21}Co_{0.37}Ni_{0.29}Mn_{0.13})_2B$	$(Fe_{0.17}Co_{0.44}Ni_{0.26}Mn_{0.13})_2B$
F	$(Fe_{0.41}Co_{0.18}Ni_{0.14}Mn_{0.27})_2B$	$(Fe_{0.39}Co_{0.26}Ni_{0.13}Mn_{0.22})_2B$	$(Fe_{0.38}Co_{0.24}Ni_{0.19}Mn_{0.19})_2B$	$(Fe_{0.28}Co_{0.36}Ni_{0.18}Mn_{0.15})_2B$	$(Fe_{0.26}Co_{0.39}Ni_{0.22}Mn_{0.13})_2B$	$(Fe_{0.25}Co_{0.42}Ni_{0.21}Mn_{0.12})_2B$	$(Fe_{0.20}Co_{0.48}Ni_{0.24}Mn_{0.08})_2B$
G	$(Fe_{0.50}Co_{0.18}Ni_{0.09}Mn_{0.23})_2B$	$(Fe_{0.44}Co_{0.24}Ni_{0.12}Mn_{0.20})_2B$	$(Fe_{0.40}Co_{0.30}Ni_{0.13}Mn_{0.17})_2B$	$(Fe_{0.33}Co_{0.33}Ni_{0.17}Mn_{0.14})_2B$	$(Fe_{0.28}Co_{0.40}Ni_{0.20}Mn_{0.12})_2B$	$(Fe_{0.24}Co_{0.52}Ni_{0.14}Mn_{0.10})_2B$	$(Fe_{0.20}Co_{0.52}Ni_{0.20}Mn_{0.08})_2B$

Series 2

	1	2	3	4	5	6	7
A	$(Fe_{0.23}Co_{0.16}Ni_{0.10}Mn_{0.51})_2B$	$(Fe_{0.22}Co_{0.25}Ni_{0.09}Mn_{0.44})_2B$	$(Fe_{0.22}Co_{0.28}Ni_{0.13}Mn_{0.38})_2B$	$(Fe_{0.19}Co_{0.33}Ni_{0.16}Mn_{0.32})_2B$	$(Fe_{0.14}Co_{0.38}Ni_{0.14}Mn_{0.34})_2B$	$(Fe_{0.12}Co_{0.44}Ni_{0.19}Mn_{0.25})_2B$	$(Fe_{0.09}Co_{0.50}Ni_{0.19}Mn_{0.22})_2B$
B	$(Fe_{0.29}Co_{0.18}Ni_{0.12}Mn_{0.41})_2B$	$(Fe_{0.27}Co_{0.23}Ni_{0.10}Mn_{0.40})_2B$	$(Fe_{0.20}Co_{0.30}Ni_{0.17}Mn_{0.33})_2B$	$(Fe_{0.22}Co_{0.26}Ni_{0.19}Mn_{0.33})_2B$	$(Fe_{0.18}Co_{0.36}Ni_{0.14}Mn_{0.32})_2B$	$(Fe_{0.17}Co_{0.42}Ni_{0.17}Mn_{0.24})_2B$	$(Fe_{0.13}Co_{0.47}Ni_{0.20}Mn_{0.20})_2B$
C	$(Fe_{0.32}Co_{0.13}Ni_{0.10}Mn_{0.45})_2B$	$(Fe_{0.28}Co_{0.21}Ni_{0.14}Mn_{0.38})_2B$	$(Fe_{0.27}Co_{0.27}Ni_{0.13}Mn_{0.33})_2B$	$(Fe_{0.25}Co_{0.28}Ni_{0.18}Mn_{0.29})_2B$	$(Fe_{0.22}Co_{0.34}Ni_{0.22}Mn_{0.22})_2B$	$(Fe_{0.21}Co_{0.37}Ni_{0.21}Mn_{0.21})_2B$	$(Fe_{0.14}Co_{0.43}Ni_{0.25}Mn_{0.18})_2B$
D	$(Fe_{0.39}Co_{0.16}Ni_{0.13}Mn_{0.32})_2B$	$(Fe_{0.36}Co_{0.16}Ni_{0.16}Mn_{0.32})_2B$	$(Fe_{0.31}Co_{0.17}Ni_{0.21}Mn_{0.31})_2B$	$(Fe_{0.27}Co_{0.23}Ni_{0.23}Mn_{0.27})_2B$	$(Fe_{0.25}Co_{0.29}Ni_{0.25}Mn_{0.21})_2B$	$(Fe_{0.21}Co_{0.36}Ni_{0.25}Mn_{0.18})_2B$	$(Fe_{0.17}Co_{0.38}Ni_{0.31}Mn_{0.14})_2B$
E	$(Fe_{0.45}Co_{0.13}Ni_{0.16}Mn_{0.26})_2B$	$(Fe_{0.39}Co_{0.18}Ni_{0.18}Mn_{0.25})_2B$	$(Fe_{0.38}Co_{0.17}Ni_{0.21}Mn_{0.24})_2B$	$(Fe_{0.30}Co_{0.23}Ni_{0.27}Mn_{0.20})_2B$	$(Fe_{0.24}Co_{0.24}Ni_{0.31}Mn_{0.21})_2B$	$(Fe_{0.24}Co_{0.28}Ni_{0.31}Mn_{0.17})_2B$	$(Fe_{0.23}Co_{0.26}Ni_{0.35}Mn_{0.16})_2B$
F	$(Fe_{0.49}Co_{0.12}Ni_{0.18}Mn_{0.21})_2B$	$(Fe_{0.46}Co_{0.16}Ni_{0.19}Mn_{0.19})_2B$	$(Fe_{0.44}Co_{0.15}Ni_{0.22}Mn_{0.19})_2B$	$(Fe_{0.39}Co_{0.19}Ni_{0.27}Mn_{0.15})_2B$	$(Fe_{0.32}Co_{0.24}Ni_{0.28}Mn_{0.16})_2B$	$(Fe_{0.26}Co_{0.22}Ni_{0.37}Mn_{0.15})_2B$	$(Fe_{0.25}Co_{0.25}Ni_{0.39}Mn_{0.11})_2B$
G	$(Fe_{0.53}Co_{0.09}Ni_{0.19}Mn_{0.19})_2B$	$(Fe_{0.53}Co_{0.11}Ni_{0.18}Mn_{0.18})_2B$	$(Fe_{0.43}Co_{0.18}Ni_{0.25}Mn_{0.14})_2B$	$(Fe_{0.41}Co_{0.14}Ni_{0.31}Mn_{0.14})_2B$	$(Fe_{0.33}Co_{0.15}Ni_{0.33}Mn_{0.19})_2B$	$(Fe_{0.28}Co_{0.19}Ni_{0.41}Mn_{0.12})_2B$	$(Fe_{0.20}Co_{0.23}Ni_{0.44}Mn_{0.13})_2B$

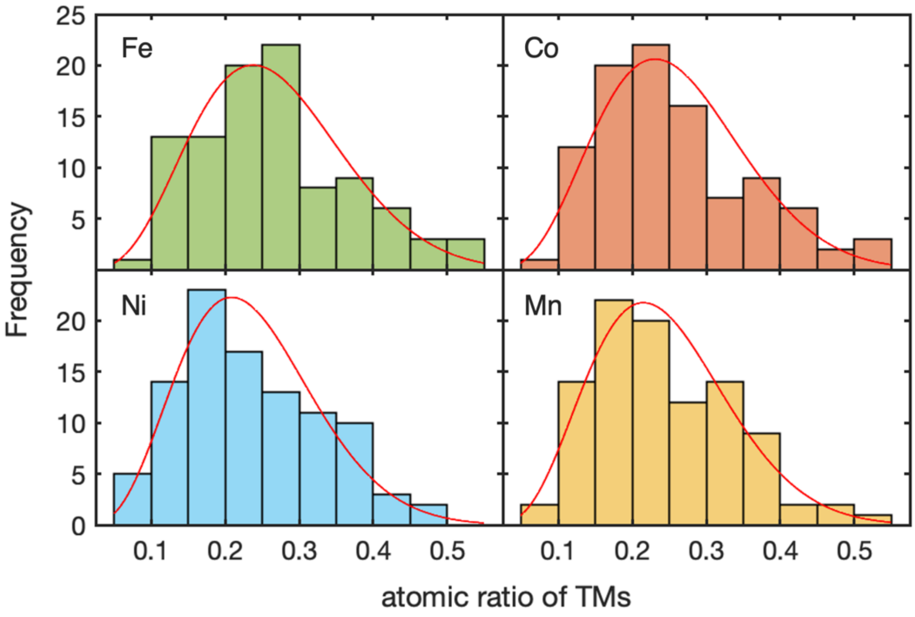

Figure S1. Histogram of the atomic ratios of transition metals in the combinatorial boride films listed in Table S1. The solid red lines are beta fits.

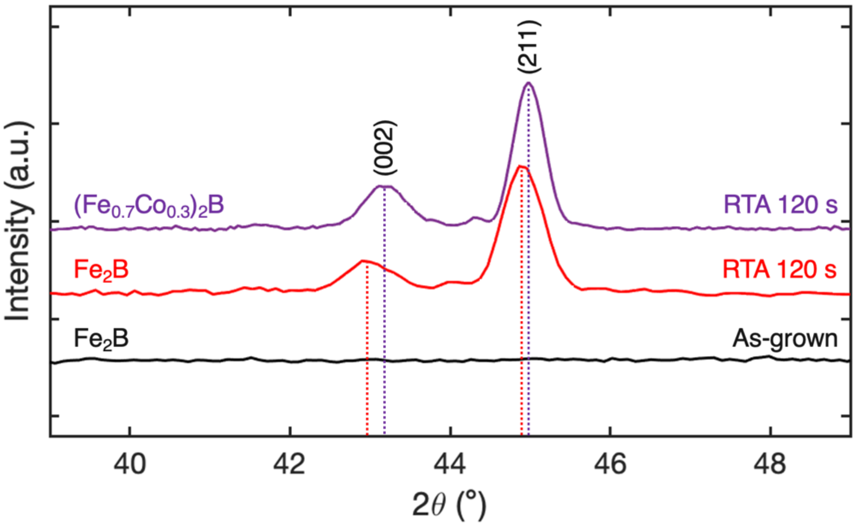

Figure S2. θ-2θ XRD scans of 20 nm as-grown and RTA-treated Fe_2B films and RTA-treated $(Fe_{0.7}Co_{0.3})_2B$ films. The RTA time and temperature was 120 s and 600 °C, respectively. The dashed lines show the positions of the measured (002) and (211) diffraction peaks.

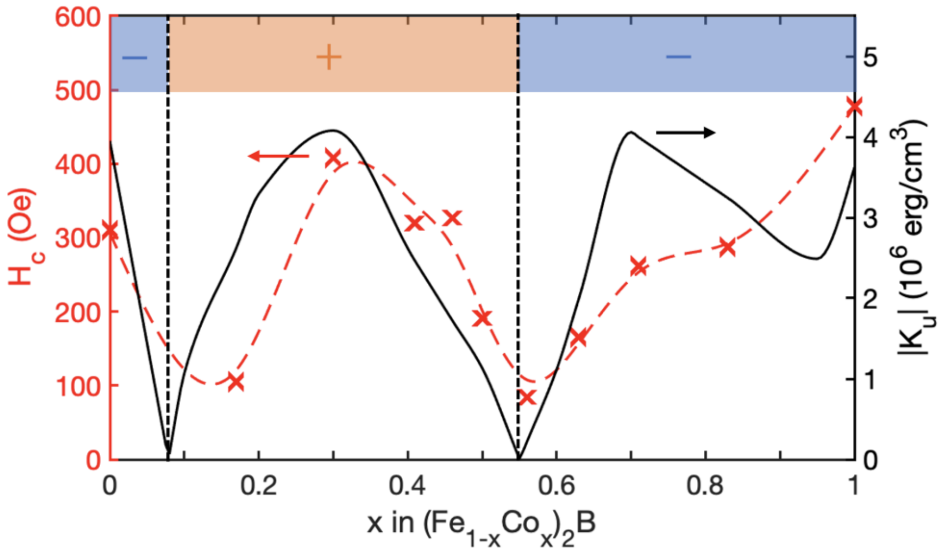

Figure S3. Coercivity H_c (red crosses) of 50 nm $(Fe_{1-x}Co_x)_2B$ films as a function of x. The dashed line is a guide to the eye. The black line is the magnitude of experimental K_u for bulk $(Fe_{1-x}Co_x)_2B$ single crystals measured at 300 K, interpolated from Iga et al.[1] The spin reorientation transitions are indicated by dashed lines and the sign of K_u is indicated at the top of the plot.

Ternary $(Fe_{1-x}Co_x)_2B$ films

Ternary $(Fe_{1-x}Co_x)_2B$ films were grown on Si/SiO_2 substrates by co-sputtering, as described in Methods, to confirm the formation of C16 structure and magnetic properties. Fig. S2 shows the θ-2θ XRD scans of 20 nm Fe_2B and $(Fe_{0.7}Co_{0.3})_2B$ films. The as-grown Fe_2B film showed no diffraction peaks, indicating a primarily amorphous structure free of C16 ordering. The amorphous structure is expected for room-temperature deposition due to the large atomic size difference between B and the TMs.[2] In contrast, both Fe_2B and $(Fe_{0.7}Co_{0.3})_2B$ films treated with RTA at 600 °C for 120 s exhibit characteristic C16 (002) and (211) diffraction peaks. The lattice parameters of the Fe_2B film are found to be a = 5.14 Å and c = 4.21 Å, close to the bulk lattice parameters of a = 5.11 Å and c = 4.25 Å.[3] The lattice parameters of $(Fe_{0.7}Co_{0.3})_2B$ are found to be a = 5.14 Å and c = 4.19 Å, compared with the bulk lattice parameters of a = 5.09 Å and c = 4.24 Å.[3b]

To investigate the magnetic properties of $(Fe_{1-x}Co_x)_2B$ films in comparison with prior work, we fabricated a series of 50 nm $(Fe_{1-x}Co_x)_2B$ films with varying Co concentration x by co-sputtering and RTA at 600 °C for 240 s. Fig. S3 shows the experimental in-plane coercivity H_c measured by VSM as a function of x, overlaid on the magnitude of the experimental K_u at 300 K for bulk $(Fe_{1-x}Co_x)_2B$.[1] Note the two spin reorientation transitions near $x = 0.08$ and $x = 0.55$, where the anisotropy crosses 0 and changes sign between negative (easy-plane) and

positive (easy-axis). The trend in H_c shows similar features to $|K_u|$. In particular, the H_c peaks locally at $x = 0.3$ coinciding with the peak in K_u, while a local minimum in H_c is found near the spin reorientation transition at $x = 0.55$. The trends in measured coercivity appear to reflect the variation in magnetocrystalline anisotropy of the C16 phase, with especially high H_c (410 Oe) near the expected peak in positive K_u. Sizeable H_c are also found in the region of high negative K_u, with the highest $H_c \approx 500$ Oe observed for Co_2B. The sizeable H_c for negative K_u may be explained by non-coherent reversal modes in the films.[4]

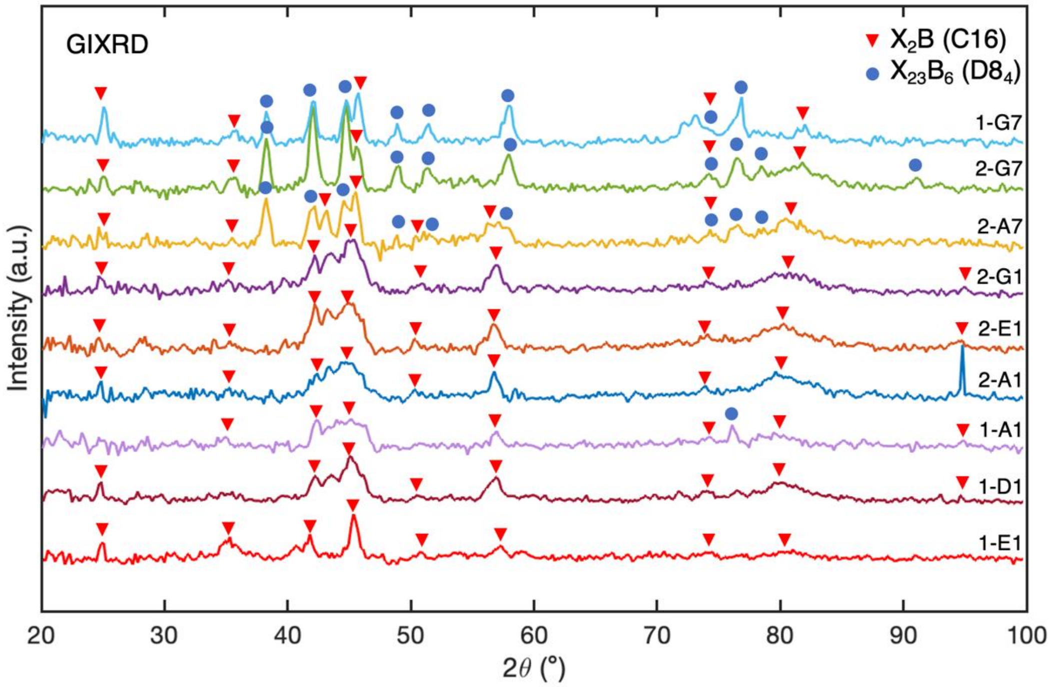

Figure S4. GIXRD scans (ω = 1°) of combinatorial $(Fe_xCo_yNi_zMn_{1-x-y-z})_2B$ samples across Series 2. The intensity is plotted on a linear scale.

XRD of combinatorial $(Fe_xCo_yNi_zMn_{1-x-y-z})_2B$

GIXRD scans ($\omega = 1°$) were carried out on selected combinatorial samples for phase analysis, as shown in Fig. S4. See Table S1 for sample compositions. All samples exhibit characteristic C16 diffraction peaks, confirming the formation of C16 phase across the combinatorial film. The GIXRD patterns generally exhibit low signal-to-noise and broad or overlapping peaks between $2\theta = 42°$ and $2\theta = 46°$, which are especially visible for samples 1-A1, 1-D1, 2-A1, 2-E1, and 2-G1. The low peak intensities suggest sparse C16 crystallites, likely within the amorphous parent matrix. The peak broadening and non-monotonic variation in peak widths throughout the pattern suggest local stresses or fluctuations in composition and lattice parameter.

Alongside the C16 phase, the Ni-rich 2-A7 and Co-rich 2-G7 samples exhibit well-defined diffraction peaks consistent with a $D8_4$ phase. $D8_4$ boride phases (e.g. $Fe_{23}B_6$, $Co_{23}B_6$, $Ni_{23}B_6$) occurring at ~20 at.% B are metastable and typically observed as secondary crystallization products during rapid solidification.[5] The appearance of this phase suggests that the total B concentration in Co-rich and Ni-rich samples may deviate from the 1:2 ratio of the stoichiometric C16 phase, thereby reducing its stability. Furthermore, the rapid cooling during RTA process may promote their stabilization.

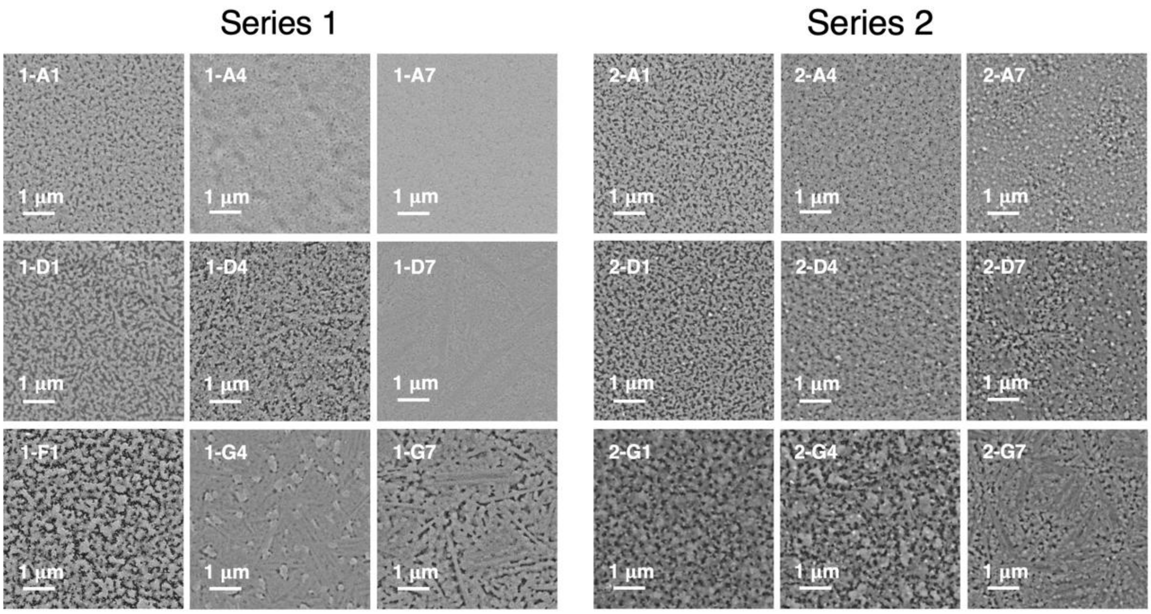

Figure S5. SEM images of selected combinatorial $(Fe_xCo_yNi_zMn_{1-x-y-z})_2B$ samples.

SEM of combinatorial $(Fe_xCo_yNi_zMn_{1-x-y-z})_2B$

To investigate the variation in microstructure among the samples, the surface morphology was imaged using scanning electron microscopy (SEM), as shown in Figure S5 for selected samples. All displayed samples in Series 2 exhibit small voids or film discontinuities. Similar morphologies can be seen in Series 1, except for samples 1-A7 and 1-D7 which remain relatively smooth. Such film break-up and void formation is known to occur during RTA due to thermal stresses, surface energy effects, and structural phase transformations.[6] These microstructural features may contribute to the high coercivities achieved in the films by promoting domain wall pinning. We note that several of the films exhibiting such voids also exhibit relatively low coercivities (1-A4, 1-G4, 1-G7, 2-A1, 2-A7, 2-D4, 2-G7 in Figure S5) suggesting that the surface morphology alone does not account for the high H_c, which still requires sizeable magnetic anisotropy. However, it should be noted that differences in the

microstructure, such as the size, shape, and density of voids, in addition to the C16 phase fraction and anisotropy, may contribute to the observed variations in H_c.

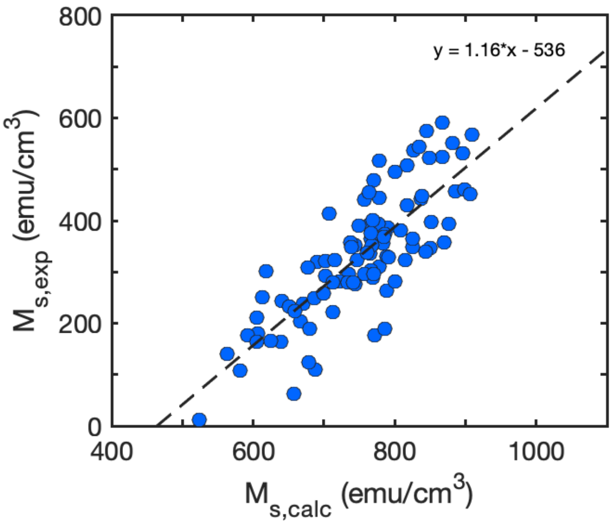

Figure S6. Experimental versus calculated saturation magnetization M_s of combinatorial $(Fe_xCo_yNi_zMn_{1-x-y-z})_2B$ samples. The dashed line is the linear least-squares fit.

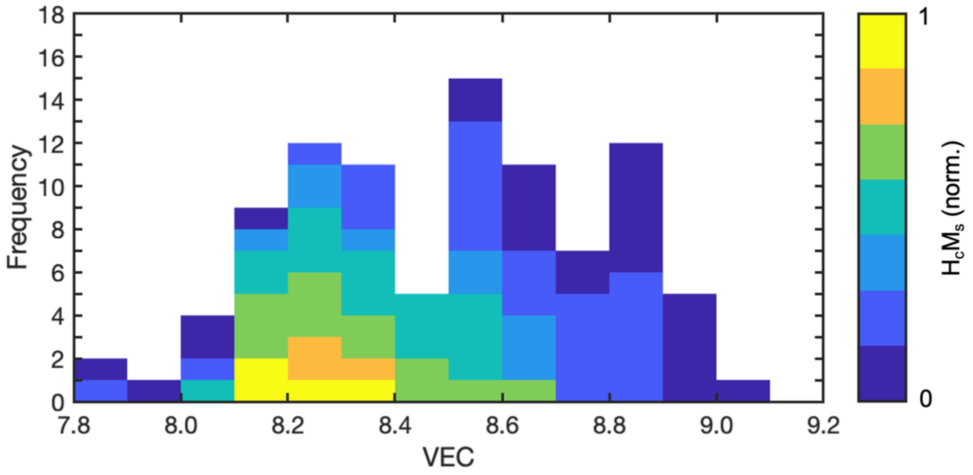

Figure S7. Histogram of the effective transition metal VEC in the combinatorial $(Fe_xCo_yNi_zMn_{1-x-y-z})_2B$ films. The effective VEC is calculated based on the EDX-estimated TM concentrations (Table S1). The bins are colored according to the product of H_c and M_s measured at 300 K, normalized to the maximum value.

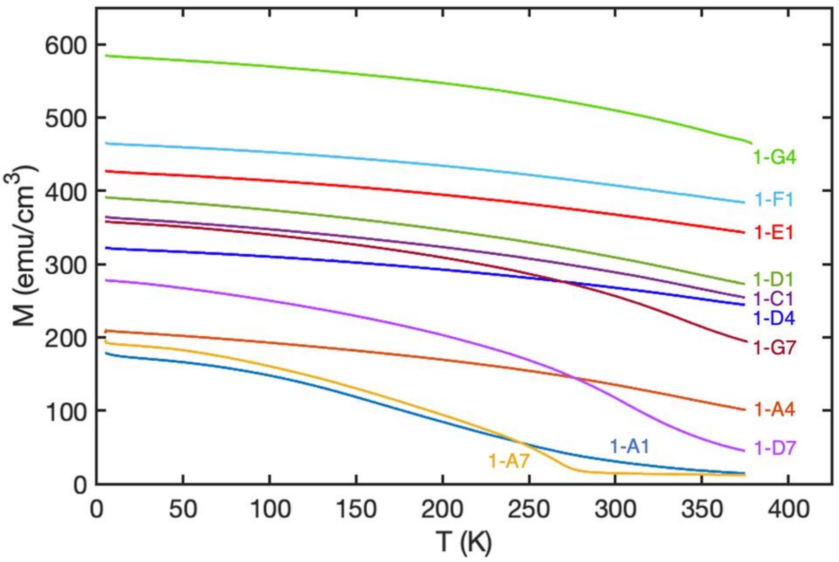

Figure S8. Temperature-dependent magnetization curves of selected combinatorial samples in Series 1. Measurements were carried out in an applied field of 200 Oe.

Temperature-dependent magnetization

Temperature-dependent magnetization measurements were carried out using SQUID magnetometer for selected samples in Series 1, as shown in Fig. S8. The films were measured while warming up from 5 K to 375 K in an applied field of 200 Oe, after field-cooling to 5 K in a field of 1 T. Samples 1-A4, 1-C1, 1-D1, 1-D4, 1-E1, 1-F1, 1-G4, and 1-G7 exhibit FM behavior throughout the measurement range, indicating thermally stable FM order with Curie temperatures (T_C) exceeding 375 K. In contrast, samples 1-A1, 1-A7, and 1-D7 each exhibit significant magnetization reductions over the temperature range, indicating FM to paramagnetic transitions below or near 375 K. The low T_C's in 1-A1 and 1-A7 are consistent with the low M_s observed in their hysteresis loops measured at room-temperature (Fig. 2a). From this, it is concluded that the thermal stability of the FM phase is sensitive to the TM composition. In particular, the Curie temperature is enhanced for high Fe concentrations, while significantly reduced to below room-temperature for high Ni or Mn concentrations.

References

[1] A. Iga, *Jpn. J. Appl. Phys.* **1970**, 9, 415.
[2] A. Inoue, *Acta Mater.* **2000**, 48, 279.
[3] a) E. E. Havinga, H. Damsma, P. Hokkeling, *J. Less Comm. Met.* **1972**, 27, 169; b) A. Edström, M. Werwiński, D. Iuşan, J. Rusz, O. Eriksson, K. P. Skokov, I. A. Radulov, S. Ener, M. D. Kuz'min, J. Hong, M. Fries, D. Y. Karpenkov, O. Gutfleisch, P. Toson, J. Fidler, *Phys. Rev. B* **2015**, 92, 062408.
[4] B. Balasubramanian, P. Manchanda, R. Skomski, P. Mukherjee, S. R. Valloppilly, B. Das, G. C. Hadjipanayis, D. J. Sellmyer, *Appl. Phys. Lett.* **2016**, 108, 152406.
[5] a) P. R. Ohodnicki, N. C. Cates, D. E. Laughlin, M. E. McHenry, M. Widom, *Phys. Rev. B* **2008**, 78, 144414; b) X. X. Wei, W. Xu, J. L. Kang, M. Ferry, J. F. Li, *J. Mater. Sci. Tech.* **2017**, 33, 352.
[6] a) S. N. Hsiao, S. C. Wu, S. H. Liu, J. L. Tsai, S. K. Chen, Y. C. Chang, H. Y. Lee, *J. Magn. Magn. Mater.* **2015**, 394, 121; b) Y.-C. Wu, L.-W. Wang, M. T. Rahman, C.-H. Lai, *J. Appl. Phys.* **2008**, 103, 07E126; c) D. A. Gilbert, J. W. Liao, L. W. Wang, J. W. Lau, T. J. Klemmer, J. U. Thiele, C. H. Lai, K. Liu, *APL Mater.* **2014**, 2, 086106; d) S. N. Hsiao, S. H. Liu, S. K. Chen, T. S. Chin, H. Y. Lee, *Appl. Phys. Lett.* **2012**, 100, 261909; e) W. B. Beeson, D. Bista, H. Zhang, S. Krylyuk, A. Davydov, G. Yin, K. Liu, *Adv. Sci.* **2024**, 11, 2308574.
